# Hyper IgE Syndrome in an Isolated Population in Israel

**DOI:** 10.3389/fimmu.2022.829239

**Published:** 2022-02-04

**Authors:** Idit Lachover-Roth, Irina Lagovsky, Atalia Shtorch-Asor, Ronit Confino-Cohen, Eyal Reinstein, Ben-Zion Garty

**Affiliations:** ^1^ Allergy and Clinical Immunology Unit, Meir Medical Center, Kfar-Saba, Israel; ^2^ Sackler School of Medicine, Tel Aviv University, Tel-Aviv, Israel; ^3^ Felsenstein Medical Research Center, Rabin Medical Center, Petach-Tikva, Israel; ^4^ Medical Genetics Institute, Meir Medical Center, Kfar-Saba, Israel; ^5^ Allergy and Clinical Immunology Unit, Schneider Children’s Medical Center, Petach-Tikva, Israel

**Keywords:** hyper IgE syndrome, HIES, ZNF341, isolated population, primary immunodeficiency

## Abstract

**Introduction:**

Hyper IgE syndromes (HIES) are a group of rare primary immunodeficiency characterized by high levels of serum IgE, cold abscesses, pulmonary infections, and eczema. ZNF341 deficiency was described in 2018 in 11 patients clinically diagnosed previously with HIES. Eight of those patients, all offspring of consanguineous couples, are from three families who live in a Muslim village in Israel which has approximately 15,000 residents.

**Objective:**

Our study aimed to evaluate the prevalence of ZNF341 mutation in the population of the village.

**Methods:**

Three hundred DNA samples of females were included in the study. The samples belong to females that were referred to the Meir Medical Center for prenatal genetic testing before pregnancy, during 2017-2019: 200 samples were from the village, and 100 samples of Muslim females were from other villages.

All samples were tested by Sanger sequencing for the ZNF341 mutation (c.904C>T, NM_001282933.1).

**Results:**

Heterozygous nonsense mutation in ZNF341 was found in ten samples (5%) of the study group compared to zero in the control group (p<0.01).

**Conclusion:**

The carrier frequency of the mutation in ZNF341 in the studied village population is 1:20. This high frequency is probably due to founder mutation and consanguineous marriages.

## Introduction

Hyper IgE syndromes (HIES) are primary immune deficiency (PID) characterized by skin abscesses, recurrent pulmonary infections, eczema, increased high morbidity and mortality and high serum IgE levels (1). HIES includes different disorders that are gathered together due to two common features: high levels of IgE (1), and an abnormal expression of signal transducer and activator of transcription 3 (STAT3) (2). STAT3 is essential for the regulation of T helper 17 (T_H_17) cells which are the primary source for interleukin (IL) 17, a pro-inflammatory cytokine involved in the protection against *Staphylococcus aureus* and *Candida* infections (3).

HIES was first described by Davis et al. in 1966, who coined the name Job’s syndrome (4). HIES was initially suspected based on the NIH score that is based on clinical and laboratory findings (5). The first genetic variant in *STAT3* gene that causes autosomal dominant (AD) HIES was discovered only four decades after the initial clinical description (6). During the last decades, other variants that cause autosomal recessive (AR) HIES were discovered in different genes (2, 7). These pathogenic variants differ in the clinical manifestation (7) probably due to different levels of influence on STAT3 expression and activation. Recently, the international union of immunological societies expert committee, updated the classification of HIES, and some of the syndromes are no longer part of the group of HIES such as Dedicator of cytokinesis 8 (DOCK8) deficiency and Tyrosine kinase2 (TYK2) deficiency, although those deficiencies influence STAT3 activity (8–11). Instead, other syndromes are now part of the HIES, include Comel- Netherton syndrome, ERBB2-interacting protein (ERBIN) deficiency, IL 6 receptor (IL6R) deficiency, IL6ST deficiency, and Loes- Dietz syndrome (11).

The following disorders are connected to the HIES syndromes and influence the activation of STAT3:

*AD STAT3 deficiency* – Also called Job’s syndrome. This is the most recognized and widespread form of HIES and the only AD form. Those patients suffer from eczema, recurrent skin *Staphylococcus aureus* infections, cold abscesses, pulmonary infections with pneumatocele, and fungal infections. They also have non- infections manifestations including failure to exfoliate primary teeth, bone fractures after mild trauma, scoliosis, and dysmorphic features that developed with time including bulbous nose and pitted skin. The laboratory findings include high levels of IgE, eosinophilia, and low level of T helper cells 17 (8).

*Phosphoglucomutase 3 (PGM3) deficiency* – This is an AR form of HIES, was first described in 2014 in patients who were previously diagnosed with HIES or severe combined immunodeficiency (12, 13). Those patients suffer from severe recurrent bacterial respiratory infections, eczema, and allergies. Non-immunological features include scoliosis, and intellectual impairment (1, 7). Like patients with other forms of HIES, those patients have elevated serum IgE, eosinophilia, but in addition, they have high IgG, and B- cell lymphopenia (7).

*IL6ST deficiency –* A homozygous variant in IL6ST, described in 2017 in one patient (14). This deficiency is characterized by bacterial infections, eczema, pulmonary infections, and abscesses, and noninfectious manifestations include retention of primary teeth, scoliosis, craniosynostosis, and high IgE levels. Two years later, 12 patients were diagnosed with a heterozygous mutation in IL6ST (15).

*IL6R deficiency –* Two patients with atopic dermatitis, recurrent skin and lung infections, sinopulmonary infections, and elevated serum IgE, were described as having a homozygous variant in IL6R (16).

*Zinc finger 341 (ZNF341) deficiency* – The latest AR form of HIES, was described in 2018 in 11 patients (eight patients with the same mutation from Israel and three others with different mutations) (2). Most of the patients fulfilled the diagnosis criteria of HIES according to the NIH clinical score (5). They present a combination of high IgE levels, severe dermatitis, skin infections, cold abscesses, recurrent pneumonia, oral thrush, and intellectual disability. Those patients receive regular preventive antibiotic treatment. Seven of the eight Israeli patients have similar clinical characteristics including skin infections with *Staphylococcus aureus* and eczema and NIH clinical score between 23-62. However, the last patient who shares with them the same mutation was five years old when examined and had only 12 points according to this scoring system, showing that some patients with this mutation might have a mild form of the disease, a finding that may encourage the study of patients with partial expression of this syndrome. All the eight patients were offspring of consanguineous couples (first-degree cousins) from three families who live in a Muslim village in northern Israel. This village habitants (approximately 15,000 persons) can be divided into several tribes by their similar family surname. The three families are from the same tribe and share the same family name although they claim they are neither related nor familiar with each other. Until lately the population of the village did not marry outside the village and the consanguinity marriage rate was high.

Our study aimed to evaluate the prevalence of *ZNF341* pathogenic variant in this relatively isolated population and to compare it to the general Muslim population in Israel.

## Materials and Methods

The study was approved by the local ethical committee of Meir Medical Center, Number in the IRB 0312-19 MMC.

Population: Three hundred DNA samples from 300 females, were obtained from the medical genetics institute in Meir Medical center. The DNA samples were collected during the years 2017-2019 from women who underwent prenatal genetic screening. The DNA samples were divided into three groups: A. 100 samples from the studied village whose surname was the same as the surname of the patients (group A). B. 100 samples from the studied village with a different surname (group B). C. 100 samples of Muslim females from other villages, in geographic vicinity. Those samples served as a control group (group C). It was taken into consideration that groups A & B can be mixed due to changing family name after marriage.

Genetic analysis: The 300 samples were tested by TaqMan^®^ SNP (single- nucleotide polymorphism) genotyping assay (Life Technologies, USA) for the ZNF341 nonsense variant (c.904C>T, NM_001282933.1) described as causing HIES. The analyses were performed on the Applied Biosystems (Step One Software v.2.3), Fast Real-Time PCR system in 96 well plates according to the manual instructions. The results were analyzed by using the setting for the SNP assay with subsequent determination of genotypes.

## Results

A heterozygous nonsense variant in *ZNF341* was found in 10 samples. Six of those samples belong to group A (6%), and the other four belongs to group B (4%). Overall, 10/200 (5%) DNA samples from females of the studied village carried the *ZNF341* nonsense variant compared to none in the control group that include samples from females from other Muslim villages, group C (p<0.01).

## Discussion

Pathogenic variants in ZNF341 (c.904C>T, NM_001282933.1) are the cause of one of the AR HIES (2). We found that the carrier frequency of this variant is 1:20 in an isolated Muslim village in Israel and is unique for this population.

The current cohort includes 100 women from this village and having the same surname as the eight patients previously described (2). Those women and the described patients are considered to belong to the same tribe. Another 100 women with a different surname from the same village were tested as well. The prevalence of the mutation was similar in both groups. However, this mutation was not found in other Muslim population living nearby in Israel.

The spectrum of HIES phenotypes is wide and some of the patients can present with non-specific clinical presentation. As already described, the ZNF341 HIES phenotype causes significant morbidity (2). As the oldest recognized patient is less than 30 years old, the life expectancy of these patients is still unknown. As the prevalence of the carriers for this variant is 1:20 and the village population is 15,000 individuals, it is estimated that there are more undiagnosed patients suffering from HIES due to the ZNF341 variant living in this village. The next step pointed toward collaboration with the primary physicians in the village is to search and diagnose those patients. Revealing those patients will help give them better treatment and can throw light on the symptoms and severity of this variant.

It is well known that isolated populations with a high consanguinity rate are at risk for a high incidence of unique genetic variants. The consanguinity rate in the investigated village is as high as 28.6% (17). This creates a convenient condition for the development of founder mutations, as the one described in the ZNF341 gene. Other unique variants were previously described in this isolated population including those causing Spinal Muscular Atrophy, non-syndromic intellectual disability, and other intellectual disabilities. Those variants are already included in the prenatal genetic screening recommendations for couples from the village.

The criteria for genetic screening recommendations of specific populations in Israel include severe genetic disease, early-onset of the disease, and high frequency of carriers in the screened population with a predicted incidence of at least 1:15,000 live births (18). The ZNF341 mutation meets the criteria for a population screening program for this specific population, and it is now included in the genetic prenatal screening recommendations by the Israeli Ministry of Health. Population screening can help early diagnosis and to consider giving early preventive antibiotic treatment. The capability to follow-up closely after those patients can help to diagnosis earlier unusual infections that affect patients with HIES, and prevent complications related to delayed diagnosis of those infections. Consequently, this can lead to reduce morbidity and improve the outcome of the patients. Just after several years of introducing early diagnosis, based in population screening for this rare disease, we can learn about the benefits of this approach.

## Data Availability Statement

Publicly available datasets were analyzed in this study. This data can be found here: https://pubmed.ncbi.nlm.nih.gov/29907690/.

## Ethics Statement 

The studies involving human participants were reviewed and approved by Ethic committee of Meir Medical Center, Israel. Written informed consent for participation was not required for this study in accordance with the national legislation and the institutional requirements.

## Author Contributions

IL-R: Substantial contributions to conception and design, analysis and interpretation of data, and drafting the article. IL: DNA analyses, revised the manuscript critically for important intellectual content. AS-S: Contribution to the design of the study, revised the manuscript critically for important intellectual content. RC-C: Revised the manuscript critically for important intellectual content. ER: Substantial contributions to conception and design of the study, revised the manuscript critically for important intellectual content. B-ZG: Substantial contributions to conception and design of the study, analysis, and interpretation of data, revised the manuscript critically for important intellectual content. All authors contributed to the article and approved the submitted version.

## Conflict of Interest

The authors declare that the research was conducted in the absence of any commercial or financial relationships that could be construed as a potential conflict of interest.

## Publisher’s Note

All claims expressed in this article are solely those of the authors and do not necessarily represent those of their affiliated organizations, or those of the publisher, the editors and the reviewers. Any product that may be evaluated in this article, or claim that may be made by its manufacturer, is not guaranteed or endorsed by the publisher.

## Abbreviations

AD, autosomal dominant; HIES, Hyper IgE syndromes; IL, Interleukin; IL6R, Interleukin 6 receptor; PID, primary immunodeficiency; STAT3, signal transducer and activator of transcription 3; T_H_17, T helper 17; ZNF341, zinc finger 341.
